# Digital contact does not promote wellbeing, but face-to-face contact does: A cross-national survey during the COVID-19 pandemic

**DOI:** 10.1177/14614448211062164

**Published:** 2021-12-07

**Authors:** Martha Newson, Yi Zhao, Marwa El Zein, Justin Sulik, Guillaume Dezecache, Ophelia Deroy, Bahar Tunçgenç

**Affiliations:** University of Kent, UK; University of Oxford, UK; Indiana University, USA; University College London, UK; Max-Planck for Human Development, Germany; Ludwig Maximilian University, Germany; Université Clermont Auvergne, LAPSCO, CNRS, France; Ludwig Maximilian University, Germany; University of London, UK; University of Nottingham, UK; University of Oxford, UK

**Keywords:** Computer-mediated communication, COVID-19 pandemic, empathy, face-to-face contact, gender, modes of contact, social interaction, wellbeing

## Abstract

With restricted face-to-face interactions, COVID-19 lockdowns and distancing measures tested the capability of computer-mediated communication to foster social contact and wellbeing. In a multinational sample (*n* = 6436), we investigated how different modes of contact related to wellbeing during the pandemic. Computer-mediated communication was more common than face-to-face, and its use was influenced by COVID-19 death rates, more so than state stringency measures. Despite its legal and health threats, face-to-face contact was still positively associated with wellbeing, and messaging apps had a negative association. Perceived household vulnerability to COVID-19 reduced the positive effect of face-to-face communication on wellbeing, but surprisingly, people’s *own* vulnerability did not. Computer-mediated communication was particularly negatively associated with the wellbeing of young and empathetic people. Findings show people endeavored to remain socially connected, yet however, maintain a physical distance, despite the tangible costs to their wellbeing.

Humans are a highly social species, needing to maintain meaningful social connections throughout their lifetimes, for both wellbeing and survival (Hrdy, 2009; Tomasello, 2014). The need for social connections becomes even more prominent when people are faced with threatening or stressful situations, like a global pandemic (Dezecache et al., 2020). However, the COVID-19 pandemic imposed a particular challenge to maintaining social connections—the minimizing of physical contact with others, widely endorsed as one of the most effective measures for reducing the spread of the virus (WHO, 2020). With the rise in computer-mediated communications such as video calls, phoning, or messaging, minimal face-to-face contact does not necessarily equate to social isolation.

This pre-registered study aims to answer two key questions central to a range of disciplinary perspectives and literatures, including sociology, public health, social psychology, and cultural studies: (1) Which modes of contact do people around the world use during a pandemic? (2) Which modes of interaction under conditions of lockdown are best for people’s mental wellbeing? We investigate these questions across a diverse set of nations by means of a survey that included measures of social interactions and modes of contact (face-to-face, video, phone, or messaging), empathy, demographic variables (age, gender, education level), and pandemic-specific factors (stringency of COVID-19 measures and COVID-19 death rates in participants’ countries/states). These results can help scholars and public health professionals target future research and interventions aimed at mitigating the negative impact of video calls, messaging, and phone calls, alongside restricted face-to-face contact, on the population’s wellbeing.

## A human need for face-to-face contact

Human wellbeing depends on having strong social connections and support (Baumeister and Leary, 1995), but does it matter *which modes* of contact we use to connect with others? When proximity is dangerous or restricted, what happens to face-to-face contact and can it be replaced by physically distant modes of communication? If so, in terms of wellbeing, can face-to-face interaction be *successfully* replaced? These questions are particularly timely with recent social distancing measures, but have been relevant at every technological milestone, from the rise of writing in Ancient Greece (Orben, 2020) to when chatting and emailing over the Internet first became part of our daily lives (Kraut et al., 1998).

We identify two schools of thought: “naturalism,” which argues that our ancestors evolved using facial and vocal cues, as well as body language, and that these are only picked up in close proximity (Daft and Lengel, 1986; Kock, 2005) and “pluralism,” which argues that computer-mediated communication is equivalent to, or even better than, face-to-face interaction due to its anonymity and promotion of self-disclosure, that is, communicating personal information (Jiang et al., 2013). Users of computer-mediated communication are able to present an optimal version of themselves in a context of their choosing (Scott and Fullwood, 2020; Walther, 1996), though the introduction of virtual reality and a range of multisensory channels has arguably started to challenge this (see Carr, 2020).

When considering how different modes of contact relate to wellbeing and the quality of interactions, we need to bear in mind the unique benefits and limitations of each mode. These tend to equate to sensory cues or levels of synchronicity. From the naturalist perspective, face-to-face interactions involve rich visual, auditory, tactile, and contextual information that helps people pick up important social cues and share intentions and emotions. However, computer-mediated communication, such as text messaging or phone calls, tends to be less rich, either lacking or with limited opportunities for eye contact, synchronous affect, and turn-taking. Developments in video messaging technology have come some way in addressing these issues. For instance, eye contact in live-video messaging not only elicits psychophysiological responses and positive affective facial reactions, as recorded videos can, but also autonomic arousal (Hietanen et al., 2020). In sum, feelings of social closeness bare a direct relationship to the quality and quantity of communication (Kraut et al., 1998; Meier et al., 2021).

In support of naturalism, feelings of social connectedness are strongest after direct face-to-face interaction, followed by video chat, then audio chat, and finally text-based messaging (Sherman et al., 2013). This is in line with findings suggesting that interacting through phone or video chat, as compared to through email or text, leads to stronger social bonding, higher enjoyment of the conversation and better information exchange between interactants (Kumar and Epley, 2021). Although text-based contact, which is most common among young people (Odgers and Jensen, 2020), produces the weakest connections, connectivity, and interaction quality through this mode can be increased with cues such as emoticons (for reviews, see Bai et al., 2019; Tang and Hew, 2019). Past research has also shown that people choose richer modes of contact (e.g. phone calls vs messages) when interacting with someone with whom they have stronger connections (Baym et al., 2004; Yang et al., 2014).

Communicating through modes that allow the transmission of non-verbal cues, be it face-to-face or computer-mediated, improves personal connections and is associated with various aspects of greater wellbeing (Goodman-Deane et al., 2016). Duration of face-to-face contact positively relates to happiness (Vlahovic et al., 2012). While social support increases positive affect regardless of the modality, life satisfaction, and low levels of loneliness are associated with face-to-face social support far more than with computer-mediated social support (Wohn et al., 2017).

Finally, although emojis may be perceived by the brain in a way that is similar to how emotion recognition takes place (Aldunate and González-Ibáñez, 2017), they and other message-based communications do not produce as much enjoyment, perception of similarity with the sender, or closeness with the sender (Sprecher and Hampton, 2017). Considering these prior studies showing computer-mediated communication to be less sensorily rich than face-to-face contact, we test whether face-to-face still had optimal effects on wellbeing when opportunities for contact were limited, or even associated with health risks, during the early phases of the pandemic.

## The value of computer-mediated communication

Computer-mediated communication allows connection with others who are not physically present. This permits the development and maintenance of geographically displaced and potentially large social networks. In support of pluralist approaches, this “hyperpersonal” experience, afforded uniquely by computer-mediated communication, can also lead to more intimate, more satisfying, long-term social connections, even surpassing those experienced through face-to-face interaction (Scott and Fullwood, 2020; Walther, 1996).

The benefits and limitations of computer-mediated communication are nuanced and depend on how the technology is used. One meta-analysis found phone calls and text-based messaging to have overall positive associations with wellbeing (Liu et al., 2019). Even feelings of intimacy, at the far reaches of the communication spectrum, which we might expect to be challenging to establish remotely, can be achieved (Lomanowska and Guitton, 2016). Meta-analytic studies have shown that moderate use of computer-mediated communication (e.g. for social and active use) is related to greater wellbeing, and that use above or below this level relates to lower wellbeing (Dienlin and Johannes, 2020).

Older people tend to benefit most from computer-mediated communication, or multimodal methods, reportedly due to configuring their interactions in a way that generates positive emotions and feelings (Chan, 2018). For younger people, passively browsing on social network sites is associated with poorer wellbeing (Dienlin and Johannes, 2020), while active usage through direct exchanges with others is related to increased wellbeing (for a review, see Verduyn et al., 2017). Given these established age-based differences in computer-mediated communication and its link to wellbeing, we investigate whether the restrictions on face-to-face contact led to increased computer-mediated communication and if so, which groups’ wellbeing benefited from this switch.

## Changes in modes of contact during the COVID-19 pandemic

Naturalist and pluralist approaches have focused on starkly binary issues, such as face-to-face versus computer-mediated communication or costs versus benefits (Bordia, 1997). However, a more nuanced question concerns flexibility within these categories: Are humans socially flexible animals when it comes to social interaction? Just how much can computer-mediated communication alleviate our need for proximity-based face-to-face interaction, and what effects might have restricted face-to-face on one hand, and extended computer-mediated communication, on the other hand, have on our wellbeing?

Early insights suggested that computer-mediated communication increased by 64% among American adults during the early stages of the pandemic (Nguyen et al., 2021). Here, frequent online gaming, social media, and emailing were all associated with weaker social bonds, while phone or video calls and text messaging were not. It is not just interactions with friends and family that have changed, but for many people, our whole words have rapidly moved on to computers, such as interactions with workmates (Sirait and Zellatifanny, 2020) or other students (Elmer et al., 2020). Being devoid of face-to-face contact can, however, have a negative effect on individuals’ wellbeing. One meta-analysis showed that the self-isolation associated with quarantine during previous global disease outbreaks was associated with adverse psychological states, particularly so for older people with smaller networks to draw upon (Brooks et al., 2020; Kim and Jung, 2020).

Data gathered from the early stages of the COVID-19 pandemic similarly showed lower wellbeing and heightened mental health issues in several countries, with potential links to stringent measures limiting face-to-face contact and/or the increase in pandemic-related death rates (e.g. Every-Palmer et al., 2020; Fitzpatrick et al., 2020 Groarke et al., 2020; Kim and Jung, 2020; Zhao et al., 2020). A large-scale UK-based longitudinal study further showed that during the COVID-19 pandemic, individuals experiencing face-to-face contact were less likely to experience depressive symptoms, as compared to those communicating through phone or video (Sommerlad et al., 2021). Extending these findings to the global scale, in this study, we examine specifically how, in the early months of the pandemic, different modes of contact impacted people’s wellbeing.

Socio-demographics and trait empathy influence how communication through different modes of contact impact people’s wellbeing. Which demographic groups were at higher risk of poor mental health and wellbeing over the pandemic remains an open question. For instance, while meta-studies have found no gender effects on wellbeing during the pandemic (Castaldelli-Maia et al., 2021; Prati and Mancini, 2021), a systematic review (Vindegaard and Benros, 2020) and a large longitudinal probability sample study in the United Kingdom found mental health had deteriorated most among young people and women (Pierce et al., 2020). Thus, examining how demographic risk factors interact with different modes of contact to influence individuals’ wellbeing has important public health relevance.

In addition to gender and age, empathy is one personal trait that may be particularly important in exploring the associations between modes of contact and wellbeing. Empathy defines one’s ability to align with and understand others’ mental states and has been associated with wellbeing during COVID-19 among Italians (Rossi et al., 2021), and appears to facilitate adherence to pandemic rules (Miguel et al., 2021; Tunçgenç et al., 2021). But how does the wellbeing of empathetic people fare in conjunction with computer-mediated modes of contact?

Pre-pandemic studies have shown that empathy is associated with a feeling of being more present in computer-mediated communications, for both men and women (Nicovich et al., 2005). As such, empaths feel more engaged in, or more connected to their computer-mediated environment. They may focus on non-verbal cues that can pose many challenges in the zoom interface (McArthur, 2021) and experience psychological distance associated with remote modes of contact (Tree et al., 2021) particularly profoundly. One potential challenge for empaths is when the edges of their mode of contact become blurred, which may invoke “mirror anxiety” from seeing their own reflection, hyper gaze from staring at a grid of staring eyes, and the development of “third skins” (Fauville et al., 2021; Nadler, 2020). The symbiosis of background, person, and technology that occurs through frequent and extended video contact, that is, a “third skin,” has been noted as being emotionally draining (Nadler, 2020). During the pandemic, the presence of third skins, including with loved ones, not only exhausts the user who seeks absent social cues, but also depersonalizes the recipient one is having contact with. We therefore included empathy as an exploratory variable, in addition to age and gender, when explaining wellbeing in relation to modes of contact.

## The present study

The high social and mental health costs the COVID-19 pandemic are anticipated to have incurred (Ghebreyesus, 2020) are likely to be experienced internationally, which we addressed by sampling 110 countries. First, we investigated which modes of contact were most popular during the pandemic (face-to-face, video, phone, messaging). We then compared rates of contact across these modes by gender, age, people working or studying from home versus outside, and by COVID-19 death rates in each region. Finally, we tackled whether face-to-face contact still promoted wellbeing under pandemic conditions. We explored the role computer-mediated communication played in the wellbeing of men and women, and younger and older people. We also investigated how empathy interacted with the different modes of contact and its effect on wellbeing.

We investigated the following, pre-registered hypotheses:

### Hypothesis 1

More people will report interacting with others through computer-mediated communication, as compared to face-to-face contact. This trend will be more salient (1) among women than men, (2) among younger than older people, (3) among those working or studying from home than those working or studying outside their home, and (4) during acceleration phases of the pandemic.

### Hypothesis 2

Wellbeing will be positively associated with higher levels of face-to-face contact. This relationship will be moderated by the vulnerability of (1) the self and (2) loved ones to contract the disease, such that individuals with high levels of face-to-face will report higher wellbeing than those with less face-to-face contact, only when levels of vulnerability are low.

The pre-registration also detailed an additional hypothesis, for which we did not acquire sufficient data to analyze. The hypothesis was that the association between face-to-face contact and wellbeing would be weaker in countries enforcing lockdown with more cultural tightness (i.e. more norm compliance) during lockdown. Unfortunately, only 6 of the 110 countries sampled had a sample size of over 100 and a tightness score available online, resulting in a lack of variation that rendered the results meaningless. Limited analyses are available through the Open Science Framework (OSF).

### Exploratory analyses

Given differences in cue richness across computer-mediated communication, we further examined how different computer-mediated modes may be associated with wellbeing. As the interpretation of social-emotional cues is a key challenge for computer-mediated communication, we were particularly interested in their interaction with empathy. In addition, we explored whether gender and age, two demographics that had main effects on wellbeing, interacted with computer-mediated modes to influence wellbeing.

## Method

### Participants

During the months of April to May 2020, data were collected from 6675 participants. The majority of participants reported completing university or postgraduate studies (83.5%) and 31.3% were current students. At the time of completing the survey, 48.1% of the participants were always working or studying from home and 16.7% were never doing so. Participants were from a total of 115 countries, but most participants were from Australia (*N* = 135), Bangladesh (*N* = 275), Canada (*N* = 105), Colombia (*N* = 76), France (*N* = 344), Germany (*N* = 221), India (*N* = 82), Italy (*N* = 112), Peru (*N* = 724), Spain (*N* = 75), Sweden (*N* = 138), Turkey (*N* = 1148), the United Kingdom (N = 1937), and the United States (N = 543).

Several exclusion criteria were applied before analysis: (1) 115 participants (*n* = 59 non-binary, *n* = 56 preferred not to disclose their gender) who reported a gender other than “man” or “woman” were excluded to their low *n* and because some of our pre-registered analyses required directly comparing men and women. However, we included additional, exploratory analyses with these participants; (2) 41 participants had missing information about the stringency of measures used in their region; (3) 32 participants had missing information about the COVID-19 deaths reported in their region; and (4) 1110 participants were neither working nor studying, and hence had missing data for the follow-up question of whether they were working or studying from home. The latter criterion was applied only to the analyses that included the “home” variable as a predictor or covariate, resulting in a reduced dataset of 5446 participants (3643 women). In analyses that did not include the “home” variable, the sample used comprised 6523 participants (4333 women) with an age range of 16–90 years and a mean age of 36.63 (*SD* = 14.26).

### Measures

The items used for this study are a subset from a larger survey, which can be accessed from the main project page on the OSF. The present survey comprised the following: (a) demographics questions, (b) the Social Network Questionnaire (Stiller and Dunbar, 2007), (c) the Warwick-Edinburgh Mental Wellbeing Scale (Tennant et al., 2007), and (d) the empathy quotient (Wakabayashi et al., 2006). Full details can be found in the Supplemental material.

### Demographics

Participants provided their age, gender (options: man, woman, non-binary, prefer not to say), education levels (none, primary, secondary, university/college, post-graduate), whether they studied or worked and, if so, whether they currently worked or studied from home. Participants were also asked their country of residence at the time of answering, which was used to obtain the rate of deaths and stringency of lockdown measures in that country or USA state. The stringency scores were obtained by taking the average of the past 15 days’ stringency indices reported in the OxCGRT database (Hale et al., 2020) as of the day participants completed the survey. Death rates were obtained through the Johns Hopkins Coronavirus Resource Center website (https://coronavirus.jhu.edu/map.html). For participants in the United States, stringency and acceleration of the pandemic were according to state. Thus, both stringency and acceleration scores represent trends within a country or state for the 2 weeks prior to survey completion.

### Modes of contact

Following prior work (Dunbar and Spoors, 1995), participants were asked to note down the first names of all the people they had a voluntary conversation with in the last 7 days and how they interacted with them (i.e. their modes of contact). Participants were specifically instructed not to include “casual contacts, such as compulsory work meetings and interactions with shop workers or professionals (e.g. doctors, police, etc.).” Multiple modes of contact were possible for each social contact, with the options being face-to-face, video chat, phone, email, message, and other. For our analyses, email and text message categories were collapsed to form a single “message” category, as they are both text-based, lacking auditory and facial cues. Each mode of contact had a small image accompanying it to reduce ambiguity (see OSF for full survey).

### Vulnerability

Participants were asked how vulnerable they thought they and someone they cared about in their household were to the coronavirus disease. Self- and household vulnerability were assessed on 100-point scales ranging from 0 = “not vulnerable at all” through 50 = “as vulnerable as an average person” to 100 = “extremely vulnerable.”

### Procedure

Participants were asked to complete an online survey prepared using jsPsych (De Leeuw, 2015). Responses were collected in 12 languages (i.e. Arabic, Bangla, Chinese, English, French, German, Hindi, Italian, Persian, Spanish, Swedish, Turkish) and shared beyond student networks to encourage a less WEIRD (Western, Educated, Industrialized, Rich, and Democratic) sample (Henrich et al., 2010). Data were collected at six time-points in 2020, of which this study uses only Phase 1. There was a 5-week window for each language in which to complete the survey, with the first language (English) published on 9 April 2020 and the last one (Hindi) on 29 April 2020.

As is common in cross-cultural research, participation was on a voluntary basis to obtain an as unbiased sample as possible across cultures and different social-economic groups in the population. For participant recruitment, snowball sampling (through existing contacts) and volunteer sampling (through calls for participation in wider networks) were employed. These revolved around student mailing lists, university press releases, calls for participation made on local and national media and blog posts in the hosting countries. In addition, advertisements were issued in target community groups and social media platforms (e.g. COVID-19 Facebook groups).

### Statistical analysis

Data regarding the mode frequencies were highly skewed, with many people reporting zero use. We therefore selected mixed effects zero-inflated negative binomial regressions for Hypothesis 1 analyses, which were conducted using R v. 4.0.4 package GLMMadaptive (Pinheiro and Bates, 1995; R Core Team, 2019). In the model, the frequency of the four modes (face-to-face, video, phone, message) was the outcome variable, participant ID was entered as a random effect variable and mode of contact, total number of contacts, and stringency were the covariates. In addition, models were fitted to examine the interaction between the mode of contact and gender, age, and home, as well as the interaction between mode of contact and death rate or stringency. In these models, the fixed effects compare the incidences of three computer-mediated modes against the reference category of face-to-face. The zero-part coefficients compare zero incidences of the computer-mediated modes against zero incidences of the reference category (face-to-face). For Hypothesis 2 and the exploratory analyses, linear regressions were conducted using JASP (v0.14) and interaction visuals were plotted using visualization data created in PROCESS v3.5.2 on SPSS v27 (Hayes, 2017; JASP Team, 2020). Hypothesis 2’s linear regression models had wellbeing as the outcome variable and face-to-face, household or self-vulnerability and their two-way interaction with face-to-face the predictor variables. Age, total contacts, stringency, gender, home, and education were included as covariates. All continuous predictors were standardized to mean = 0 and standard deviation = 1 throughout. The dataset was re-standardized prior to analyses including “home” as a predictor, after excluding participants with missing data on this variable.

In the exploratory analyses, we conducted several multilinear regressions with wellbeing the outcome variable, and empathy and the four modes predictors. We also investigated how the four modes interacted with empathy, age, and gender, respectively. Age, total contacts, stringency, gender, home, and education were included as covariates.

## Results

### Hypothesis testing

#### Computer-mediated communication is more common than face-to-face during the pandemic

Overall, 73.7% of participants reported zero face-to-face communications in the week prior to completing the survey. Using Kendall’s τ_b_, face-to-face correlated with video (τ_b_ = .13), phone (τ_b_ = .22), and message (τ_b_ = .19), *p* s < .001, though the correlations among the three computer-mediated modes were stronger (τ_b_ > .35, *p*s < .001). To explore computer-mediated communication use in relation to face-to-face, we used negative binomial models with mode frequency the outcome, mode type the predictor, and stringency and total contacts (the number of personal contacts an individual had outside of their household in the last week) as covariates. First, in support of Hypothesis 1, we found that most people interacted through computer-mediated communication rather than face-to-face (*p* s < .001, Table 1). Participants were significantly more likely to report zero face-to-face contact than they were to report zero contact through computer-mediated communication (*p*s < .001). People were 216% more likely to message, 117% more likely to use video, and 86% more likely to use phone contact than face-to-face contact (see Table SI1 in the Supplemental material).

**Table 1.** Frequency of modes of contact, fixed effects, and zero-part coefficients.

Reference: face-to-face	Estimate	EXP^a^	*SE*	*z*	*p*
Fixed effects					
Messaging	1.15	3.16	0.03	38.09	<.001
Phone	0.62	1.83	0.03	19.90	<.001
Video	0.77	2.17	0.03	24.74	<.001
Total contacts	0.78	2.18	0.01	65.71	<.001
Stringency	−0.09	0.91	0.01	−7.68	<.001
Zero-part coefficients^b^					
Messaging	−1.67	0.19	0.10	−17.42	<.001
Phone	−1.89	0.15	0.11	−17.12	<.001
Video	−1.74	0.18	0.11	−16.02	<.001

SE: standard error.

a As the model used a log-link, that is, the log of the outcome is linearly related to the covariates, we include an EXP column to help interpret the model, whereby EXP reflects exponentiated coefficients.

b The zero-part coefficients represent how much each variable had “0” as a response, in relation to how much the reference category (face-to-face) had “0” as a response, that is, participants were significantly less likely to report not having used computer-mediated communication at all in relation to having had zero face-to-face contact.

To examine whether these relatively low levels of face-to-face contact were especially more salient in certain demographic groups or contexts, we added gender, age, the “home” variable (i.e. describing whether people worked/studied from home) and death rate as interaction terms to the mode types. In the death rate interaction model, we also included age, gender, and home as covariates. Regardless of gender (*p* = .732) or age (*p*s > .109), people reported similar amounts of face-to-face contact. Pairwise comparisons revealed that relative to face-to-face contact, women relied on messaging and video more than men, and older age groups relied on phone use more than the youngest age group (Table SI3 in the Supplemental material). As predicted, people staying at home used computer-mediated modes more than face-to-face, as compared to people working or studying outside the home (*p* < .001) (see Table 2).

**Table 2.** Modes of contact by staying home, fixed effects.

Variable	Estimate	EXP	*SE*	*z*	*p*
Messaging	0.85	2.34	0.07	12.26	<.001
Phone	0.40	1.49	0.07	5.50	<.001
Video	0.50	1.65	0.07	6.83	<.001
Home (sometimes)	−0.04	0.96	0.09	−0.47	.637
Home (yes)	−0.37	0.69	0.08	−4.63	<.001
Total contacts	0.77	2.16	0.01	60.64	<.001
Stringency	−0.09	0.91	0.01	−6.62	<.001
Message × home (sometimes)	0.06	1.06	0.09	0.67	.503
Phone × home (sometimes)	0.01	1.01	0.10	0.15	.883
Video × home (sometimes)	−0.01	0.99	0.10	−0.11	.913
Message × home (yes)	0.49	1.63	0.08	5.92	<.001
Phone × home (yes)	0.35	1.42	0.09	4.03	<.001
Video × home (yes)	0.49	1.63	0.09	5.63	<.001

SE: standard error. Reference categories are Face to Face contact and Home (no), that is, mode values are in relation to face-to-face and Home values are in relation to not staying home.

Finally, as predicted, the model with death rates as the moderator showed that when the pandemic was escalating (i.e. death rates were increasing), people were more likely to use computer-mediated communication as compared to face-to-face (*p*s < .001, Table 3). As the pandemic escalated, people were especially more likely to report zero face-to-face contact compared to reporting zero computer-mediated communication (*p*s < .001, SI). We re-ran the analysis, including stringency instead of death slope and found that death slope was a more powerful moderator, which may suggest that people were responding to the threat of the pandemic somewhat independently from state or national restrictions (see Table SI7 in the Supplemental material).

**Table 3.** Modes of contact in relation to face-to-face during peak restrictions and easing of lockdown acceleration and deceleration phases of the pandemic (death slope), fixed effects.

Reference: face-to-face	Estimate	EXP	*SE*	*z*	*p*
Messaging	1.13	3.09	0.03	34.70	<.001
Phone	0.61	1.84	0.03	17.86	<.001
Video	0.74	2.09	0.03	21.66	<.001
Death slope	−0.14	0.87	0.03	−4.20	<.001
Total contacts	0.72	2.05	0.01	56.95	<.001
Gender	0.27	1.31	0.03	9.86	<.001
Age	−0.03	0.97	0.01	−2.69	.007
Home (sometimes)	0.02	1.02	0.04	0.42	.677
Home (yes)	0.04	1.04	0.03	1.36	.173
Education	0.08	1.08	0.01	7.85	<.001
Message × death slope	0.27	1.31	0.03	7.87	<.001
Phone × death slope	0.13	1.14	0.04	3.81	<.001
Video × death slope	0.31	1.36	0.03	8.96	<.001

SE: standard error.

#### Despite risks to oneself, face-to-face contact is associated with wellbeing during the pandemic

A paired-samples *t*-test revealed that people tended to perceive loved ones in their household as more vulnerable to COVID-19 (*M* = 55.48, *SD* = 22.92) than they did themselves (*M* = 45.49, *SD* = 20.64), *t*(4831) = 30.34, *p* < .001. To investigate the effects of face-to-face contact and self and loved ones’ vulnerability (Hypothesis 2), we ran two separate linear regressions with wellbeing as the outcome variable. The two vulnerability variables were treated separately due to the strong correlation between them (*r* = .46, *p* < .001). In the first model, we included face-to-face, self-vulnerability, and their two-way interaction as predictors, with age, total number of contacts, stringency, gender, staying home, and education as covariates. In the second model, we replaced self-vulnerability with household vulnerability. In both models, we found that people with more face-to-face contact tended to have greater wellbeing (self-vulnerability model, β = .06, *SE* = 0.07; household vulnerability model, β = .03, *SE* = 0.05; *p*s < .001). There was no interaction between face-to-face and self-vulnerability (*p* = .106, SI), whereas face-to-face significantly interacted with household vulnerability (β = −.04, *SE* = 0.07, *p* = .027), such that people with more face-to-face contact who also perceived their household members as low-risk to the disease had especially greater wellbeing (see Tables SI8–9 in the Supplemental material).

### Exploratory analyses

To examine the computer-mediated modes and empathy’s (*M* = 18.21, *SD* = 3.31) associations with wellbeing, we ran four models, one for each mode of contact, with wellbeing the outcome variable and each mode’s interactions with empathy quotient (EQ) as the predictor variables. We repeated this process for each mode’s interactions with both age and gender in further models. First, we conducted a simple regression with EQ and the four modes as predictor variables. We continued to use the same covariates as in Hypothesis 2 (age, total number of contacts, stringency, gender, staying at home, and education).

**Figure 1.**
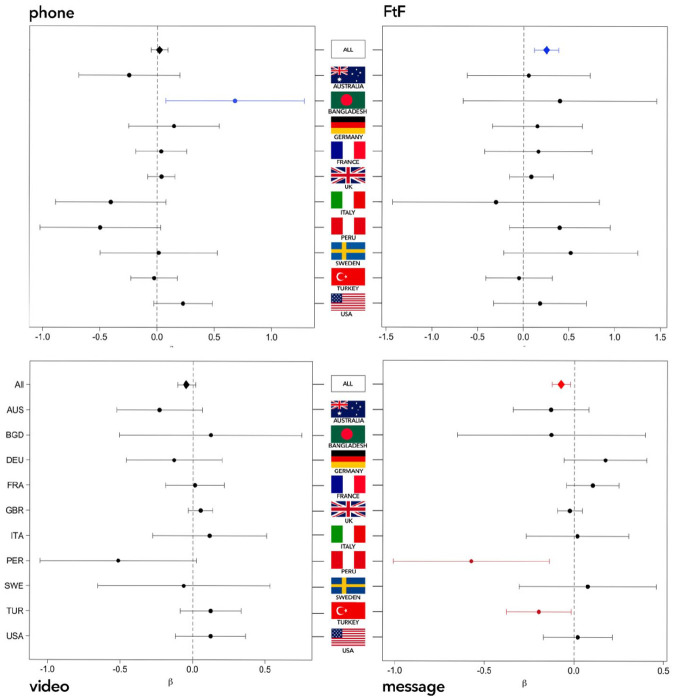
Frequency of mode of contact in the last 7 days (IV) and wellbeing score (DV) of 10 nations. The 10 countries with the largest samples (*n* > 100) are represented.

Overall, only face-to-face contact was positively associated with wellbeing (β = .06, *SE* = 0.07, *p* < .001). Messaging was associated with lower wellbeing (β = −.04, *SE* = 0.03, *p* = .047). Phone and video use did not have significant associations with wellbeing (*p*s > .912). Figure 1 provides a visualization of how each mode of contact is associated with wellbeing in the 10 countries with the largest sample sizes in our database. As can be seen, there were substantial differences between the associations of modes and wellbeing across nations. For instance, messaging had a particularly negative association with wellbeing in Peru (β = −.57, *SE* = 0.22, *p* = .010), which may have driven results. In contrast, phone calls had a particularly positive association with wellbeing in Bangladesh (β = .68, *SE* = 0.31, *p* = .028). Our exploratory model further revealed main effects of empathy, gender, age, stringency, and total contacts, such that people with higher EQ (β = .11, *SE* = 0.06, *p* < .001), men (β = .09, *SE* = 0.13, *p* < .001), older people (β = .18, *SE* = 0.07, *p* < .001), those living under more stringent polices (β = .16, *SE* = 0.06, *p* < .001), and those with more contacts (β = .06, *SE* = 0.09, *p* = .006) tended to report greater wellbeing.

The only significant mode × empathy interaction was found for video contact (video: β = −.06, *SE* = 0.03, *p* = .001, for all other modes: *p*s > .07). People who scored highly for empathy and used more video contact had lower wellbeing scores than empaths who used less video contact. There were no gender × mode interactions, suggesting that face-to-face and computer-mediated contact were associated with men and women’s wellbeing similarly (*p*s > .387). Significant mode × age interactions were found for all computer-mediated modes, such that younger people who used lots of video, phone, or messages had lower wellbeing compared to older people or young people who used computer-mediated communication less (age × video: β = .04, *SE* = 0.03, *p* = .010; age × phone: β = .04, *SE* = 0.03, *p* = .022; age × message: β = .04, *SE* = 0.02, *p* = .024). There was no face-to-face × age interaction, such that the relation between face-to-face contact and wellbeing was equal across age groups (*p* = .863).

Finally, we re-ran our main analyses with the small, non-binary sample (*n* = 59). Hypothesis 1, that computer-mediated communication was more common than face-to-face during the first pandemic lockdown, was supported (Table SM20 in the Supplemental material). Hypothesis 2, that wellbeing was more positively associated with face-to-face than computer-mediated communication was not supported—none of the modes of communication positively predicted wellbeing (Table SM21 in the Supplemental material). There were no face-to-face × vulnerability interactions in the non-binary subsample (Tables SM22–23 in the Supplemental material).

**Figure 2.**
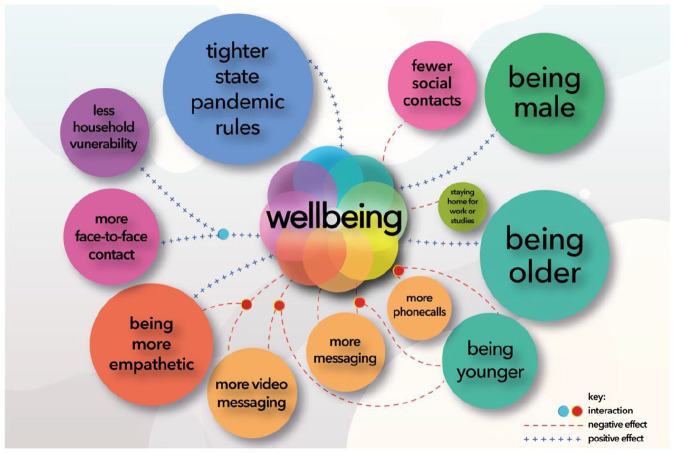
Qualitative representation of factors influencing pandemic wellbeing in our cross-national sample. Circle sizes reflect approximate effect sizes (β values). Education was also included as a covariate but was consistently non-significant (*p*s > .736). Staying at home had a consistently marginal effect on wellbeing (*p*s < .077).

Overall, our main and exploratory analyses reveal that wellbeing is positively associated with face-to-face contact, while being negatively associated with computer-mediated communication. A qualitative representation of how all these factors, and their associations with demographic and pandemic-specific variables (e.g. vulnerability to the disease, stringency of restrictions) can be seen in Figure 2.

## Discussion

This study examined which modes of contact were used in the early phases of the pandemic, and how they related to people’s wellbeing. The results from our global sample reveal that people reported more computer-mediated communication than face-to-face. This trend was consistent across age groups, for both men and women, and regardless of how the participants’ countries responded to the pandemic. It was only when people fully committed to staying at home for work or study, rather than sometimes, that they reported significantly more computer-mediated communication than face-to-face contact, as compared to people who worked or studied outside the home.

We also found that the death slope (i.e. recent increases in the number of deaths in one’s state or nation of residence, not just the number of deaths) was a stronger moderator than national stringency of pandemic measures (e.g. restrictions on travel and interaction). In support of previous literature, this may suggest that the general public follow threat cues gleaned from friends and family or the media, above and beyond following the rules set out by governments (Dezecache et al., 2020; Tunçgenç et al., 2021).

### Computer-mediated communication, age, and wellbeing

Among the computer-mediated modes we examined, wellbeing was negatively associated with messaging and had no main associations with phone or video contact. The negative association between messaging and wellbeing offers some support for the naturalist approach (Daft and Lengel, 1986; Kock, 2005). Messages lack the crucial cues that express emotions and facilitate high-quality social interactions and bonding among people, such as mutual eye gaze, shared affect, physical touch, and tone of voice. Equally, the early stages of the pandemic may have demanded a particularly acute need for social contact that surpassed normal needs.

Despite the risks and state restrictions, consistent with our hypothesis, face-to-face contact continued to be positively associated with wellbeing. This was especially the case for individuals who did not perceive any loved ones in their household to be vulnerable to COVID-19. Even people who perceived themselves as vulnerable to the disease still appeared to benefit from face-to-face contact. This demonstrates how other people, particularly one’s close social circle, continue to be paramount in our social and personal lives (Tunçgenç et al., 2021).

The importance of physical interaction, as identified in our study—at a potential cost to personal security (i.e. law breaking) or health (i.e. potential infection or death)—is likely to resonate with scholars across disciplines, from psychology to public health. At a more nuanced level, this finding also helps further inform long-standing debates around the relationship between computer-mediated communication and face-to-face interaction (Bordia, 1997; Carr, 2020).

This research highlights the significance of age regarding levels of use and the effects of computer-mediated communication. For instance, older people tended to use the phone more than younger people (16 was the minimum age in our study). We also found that age interacted with computer-mediated communication, such that younger people’s wellbeing fared particularly badly with high levels of video, phone, or messaging. All age groups appeared to benefit equally from face-to-face contact.

Previous research has suggested that although the link between computer-mediated communication and wellbeing is generally considered to be relatively weak, older people tend to benefit most from computer-mediated communication, perhaps simply due to their engaging in positive behaviors through these modes, for example, more intimate or deeper interactions (Chan, 2018). In contrast, excessive use among children (aged 9–12) is associated with low wellbeing (Bruggeman et al., 2019). Although our study only included young people (16+ years) and not children, the trend supports the wider literature.

These findings have potential implications for future research into the distance learning many teenagers around the world have participated in for several months, often for many hours a day. The adverse effects of video-based learning for young adults is perhaps magnified by the adverse effects on wellbeing that home schooling has been found to have on parents (Lades et al., 2020). We would encourage future research to consider younger people’s wellbeing in relation to computer-mediated communication, especially over extended periods.

### Empathy, gender, and culture

Despite video contact including most of the audio and visual information offered by face-to-face contact, it was still not positively associated with wellbeing in its own right. In fact, high levels of video contact were related to poorer wellbeing among people with high empathy scores. This could simply be because video is still an emerging computer-mediated mode that relies on stable Internet connections, which can be particularly challenging in rural areas and in developing economies. As such, synchronicity of audio-visual cues in video communications may be compromised. Even with stable Internet connections, video contact still lacks physical presence and shared context, as do other computer-mediated modes, which may make such interactions frustrating for particularly empathetic people.

The moderating effect of video contact on the relationship of empathy with wellbeing, may also relate to recent literature on “Zoom fatigue.” For instance, third skins manifest as a symbiosis of background, person, and technology, depersonalizing the individual one is having contact with (Nadler, 2020), and thus potentially depersonalizing oneself as a passive image in the process, consumed by the on-screen partner. During the pandemic, the presence of third skins, including with loved ones, not only exhausts the user who seeks absent social cues, but also depersonalizes the recipient one is having contact with. This may be particularly intense for empaths.

Perhaps as a result of this pervasive alienation from “third skins” and “Zoom fatigue,” there are reports of young people becoming less empathetic during the pandemic and with fewer opportunities for prosocial action, for example, in the Netherlands (van de Groep et al., 2020). The same study also found high levels of resilience, which in turn is often associated with wellbeing (Sanders et al., 2015). Drops in both empathy and opportunities for prosociality are negatively associated with socio-emotional development. We suggest that interventions to reduce reliance on computer-mediated modes, or research into approaches that will reduce its adverse effects on young people, is essential regardless of the pandemic.

We also found that men consistently reported greater wellbeing than women, though there were no interaction effects that could suggest how men’s versus women’s modes of contact were differentially associated with wellbeing. Research with Spanish participants found men’s wellbeing was greater than women’s only for people below the age of 55 (Matud et al., 2019). Masculinity (e.g. independence, assertiveness, strength, individualism, or ambition) was associated with greater wellbeing for both men and women, while femininity (e.g. empathy, tenderness, warmth, or the need for affiliation) was negatively associated with wellbeing for both men and women. Indeed, gender roles became more pronounced during the pandemic (Rosenfeld and Tomiyama, 2021), but further research is required to understand the knock on effects this would have had on feelings of masculinity, femininity, and subsequent wellbeing levels.

Although the countries in our dataset differed in terms of access to and use of technology—and experiences of COVID-19—we found a little significant variation that diverged from whole sample effects. In Bangladesh, phone calls were more associated with better wellbeing than any other region in the world. This may relate to the accessibility of telephone networks compared to poorer availability of 3G or WiFi that could hinder video messaging, as well as challenges around privacy with shared devices (Ahmed et al., 2017). The global trend observed in relation to the negative link between messaging and wellbeing was especially pronounced in Peru and Turkey. This may relate to participants from these relatively poorer nations being more familiar with sole or majority reliance on face-to-face interaction, compared to participants from the relatively richer nations, which amplified national dissatisfaction with messaging.

### Limitations

First, we did not collect information from participants about the content or valence of their interactions. It may be that exchanges through messages focused more on sharing pandemic-related news and thus negatively affected wellbeing, while face-to-face interactions were more varied in content, co-involving other activities such as walking in nature, which may have positive effects on wellbeing above and beyond the mode of contact. Relatedly, future research should investigate *who* participants contacted, and through which mode. Although our design eliminated obligatory interactions (e.g. with medical professionals or work meetings), research into the strength of social ties among interactants would be beneficial, as interacting with close others versus acquaintances may impact wellbeing differently—even when using the same mode of contact (Burke and Kraut, 2016). Finally, experimental research is required to disambiguate the direction of the associations between modes of contact and wellbeing. For instance, did frequent messaging result in worse wellbeing, or did people with lower wellbeing message more because they were trying to reach out?

Although we included a text-message icon for the mode titled “message,” there is potential ambiguity in the title, which could have led some participants to include audio messages in this category, rather than the “other” category. Furthermore, we chose not to distinguish between instant messaging (IM), texting, and email due to their low sensory richness and potential ambiguity around their synchronicity in the real world. Although IM and texting are similar in richness, texting is done on private devices and often with closer contacts, while IM is done through software using various devices. In turn, emails may be considered to require higher effort and be less synchronous than either IM or texting. Investigating social media platforms and online communities alongside more traditional forms of computer-mediated communication could also prove fruitful in developing a more complete understanding of how computer-mediated communication impacts wellbeing across generations.

Our sample had a disproportionate number of women, and people who had completed higher education, an issue we partially addressed by adding these variables as covariates in all analyses. In future research, more detailed analyses of varied samples including people with different ethnicities and/or socio-economic status will be informative given how the pandemic exacerbated existing social inequalities, including its effects on mental health and wellbeing (Gauthier et al., 2021; Otu et al., 2020). Further demographics, such as participants’ household structure, childcare, and marital status will also likely have had an impact on social interaction *outside* the home, as well as on individuals’ wellbeing.

We collected substantial data from countries that are typically considered to be less WEIRD in the psychology literature, and underrepresented in the computer-mediated communication literature, especially from Peru and Bangladesh. Our research is better framed within the WILD paradigm, that is, research that is Worldwide, In Situ (contextually relevant), Local (informed by local culture), and Distinct (beyond student participants; Newson et al., 2021). Although the survey was conducted online, limiting its ecological validity (In Situ), we recruited from a Worldwide and Distinct cohort (geographically varied, beyond student populations). We worked as a multi-national team to ensure that local information would make the survey both accessible and meaningful to as many populations as possible. Future research will benefit from studies focused on specific regions or regional comparisons, and especially by including a wider sample from the Global South.

Our hypothesis focused on gender-based differences between men and women, but since we collected data from non-binary people, albeit from a relatively small sample, we provide the analyses concerning non-binary people in the Supplemental material. We note that associations between modes of contact and wellbeing among minority groups may diverge from our results. For example, we found no positive association between face-to-face contact and wellbeing among non-binary people. This is something that should be considered in further research, especially regarding ethnic minorities and members of the lesbian, bisexual, gay, transgender, queer (LBGTQ+) communities. For instance, American sexual minorities have reported more distancing and worry around COVID-19, as well as more computer-mediated communication use in some research (Baumel et al., 2021). We also encourage future research to analyze longitudinal data to help understand how modes of contact changed as people’s understanding of the virus changed, and their personal limits of social disconnectedness were reached.

## Conclusion

As a highly social species, human beings are able to adapt their social interactions to online modes. Nonetheless, in support of the naturalist approach, we do not appear to have the social flexibility to meet all our social needs online, as face-to-face remained the only mode of contact associated with higher levels of wellbeing. In turn, increased use of computer-mediated communication did not appear to benefit wellbeing and, among young people, was a negative factor. Young people reported no more face-to-face contact in relation to their computer-mediated communication, than did older people, so previous attempts to blame young people’s disregard for social distancing rules appears misplaced. We argue that during this time of health insecurity, when speaking with social networks face-to-face was restricted, people endeavored to be physically, but not socially isolated—despite clear costs to their mental wellbeing.

## Supplemental Material

sj-docx-1-nms-10.1177_14614448211062164 – Supplemental material for Digital contact does not promote wellbeing, but face-to-face contact does: A cross-national survey during the COVID-19 pandemic Click here for additional data file. Supplemental material, sj-docx-1-nms-10.1177_14614448211062164 for Digital contact does not promote wellbeing, but face-to-face contact does: A cross-national survey during the COVID-19 pandemic by Martha Newson, Yi Zhao, Marwa El Zein, Justin Sulik, Guillaume Dezecache, Ophelia Deroy and Bahar Tunçgenç in New Media & Society
