# Supplementary material for: Digital contact does not promote wellbeing, but face-to-face contact does: A cross-national survey during the COVID-19 pandemic
Source: New Media Soc. 2021 Dec 7;26(1):426–49. doi: 10.1177/14614448211062164 (PMC10758341; doi:10.1177/14614448211062164)
Supplement: sj-docx-1-nms-10.1177_14614448211062164 – Supplemental material for Digital contact does not promote wellbeing, but face-to-face contact does: A cross-national survey during the COVID-19 pandemic [file sj-docx-1-nms-10.1177_14614448211062164.docx]

**Supplementary Information**

Table of Contents

[Methods 1](#_Toc78799772)

[Total Contacts and Modes of Contact 1](#_Toc78799773)

[Wellbeing 1](#_Toc78799774)

[Empathy Quotient (EQ) 2](#_Toc78799775)

[Additional analyses 1](#_Toc78799776)

[Checking participants who reported zero contacts 1](#_Toc78799777)

[Comparing Alternate Modes of Contact: Messaging, Phone, and Video (H1) 1](#_Toc78799778)

[Gender and Age Have No Significant Effect on Modes of Contact 2](#_Toc78799779)

[Death Slope and Stringency Analyses 2](#_Toc78799780)

[Analysing Low Stringency Participants Only (H1) 2](#_Toc78799781)

[Replacing Death Slope for Stringency 4](#_Toc78799782)

[H2 Self- and Household Vulnerability 5](#_Toc78799783)

[Analyses within the Non-binary Sample 8](#_Toc78799784)

[SI References 1](#_Toc78799785)

## Methods

### Total Contacts and Modes of Contact

The total number of contacts outside of one’s household was identified using Dunbar & Spoors’ Social Network Questionnaire (1995). We first asked participants to enter the first names of people they had voluntarily had a conversation with in the past seven days. For each social contact a participant identified, they were asked to indicate whether this contact was inside or outside of their household. The entered names were not retained but used to extract the total number of contacts outside of one’s household. Concerning modes of contact, the ‘other’ category was not analysed in this study. There were no participant-level exclusions due to this, as there were no participants who only selected the ‘other’ category without selecting at least one other mode of contact.

### Wellbeing

Participants rated their wellbeing over the previous week using the 7-item Warwick-Edinburgh Mental Well-being Scale (Tennant et al., 2007). The Persian version of this scale was not available on the scale’s official website and was therefore translated and back-translated by native speakers proficient in English before use. Items were answered on a 5-point Likert type scale with the response options: 1 = None of the time, 2 = Rarely, 3 = Some of the time, 4 = Often, 5 = All of the time. This is a single-factor scale that covers affective-emotional, cognitive-evaluative and psychological aspects of wellbeing. A participant’s wellbeing score was obtained by adding up the scores of each item and converting this raw score to the metric score following the conversion table proposed by the scale developers (Stewart-Brown et al., 2009). Total score on the scale could range between 7 and 35, with higher scores indicating better positive wellbeing. Population norm studies conducted with this scale show the mean wellbeing score to be 23.6 (23.5–23. 7) for English adults aged 16 and over (Taggart, Stewart-Brown & Parkinson, 2015).

‘I’ve been…

1. feeling optimistic about the future
2. feeling useful
3. feeling relaxed
4. dealing with problems well
5. thinking clearly
6. feeling close to other people
7. able to make up my own mind about things’

### Empathy Quotient (EQ)

EQ was measured with the shortened, 15-item version (Muncer & Ling., 2006) of the empathy quotient originally developed by Baron-Cohen and Wheelwright (2004). Each item was rated on a 4-point Likert scale (1= ‘strongly disagree’ to 4 = ‘strongly agree’). Scoring was according to Baron-Cohen and Wheelwright (2004), whereby participants scored 1 point for recording the empathetic behaviour mildly, 2 points for strongly, and 0 points for not at all. A total score was obtained by summing up all the scores, and hence had a range between 0 and 30, with higher scores indicating more empathy.

| *How strongly do you agree or disagree with the following statements? There are no right or wrong answers, or trick questions.*  [Answer options: 1 = Strongly disagree, 2 = Slightly disagree, 3 = Slightly agree, 4 = Strongly agree] |
| --- |
| 1. I am good at predicting how someone will feel |
| 1. I am quick to spot when someone in a group is feeling awkward or uncomfortable |
| 1. I can sense if I am intruding, even if the other person doesn't tell me |
| 1. I can tune into how someone else feels rapidly and intuitively |
| 1. I can easily work out what another person might want to talk about |
| 1. I find it difficult to explain to others things that I understand easily, when they don't understand it first time |
| 1. I find it hard to know what to do in a social situation |
| 1. Friendships and relationships are just too difficult, so I tend not to bother with them |
| 1. I often find it difficult to judge if something is rude or polite |
| 1. I don't tend to find social situations confusing |
| 1. I really enjoy caring for other people |
| 1. If I say something that someone else is offended by, I think that that's their problem, not mine |
| 1. Seeing people cry doesn't really upset me |
| 1. I usually stay emotionally detached when watching a film |
| 1. I tend to get emotionally involved with a friend's problems |

## Additional analyses

### Checking participants who reported zero contacts

To check whether the high reporting of zero contact might be a data quality issue, we checked for whether a pattern of “lazy responding” was present in the way these participants responded to the other questions as well. To do that, we focussed on another scale in the survey, the EQ scale, which had 15 Likert-type items presented on the same screen at once. We reasoned that if a participant skipped the social contacts question out of laziness, then they would be more likely to repeatedly choose the same response in this long EQ scale as well.

First, we identified participants who reported zero contacts (*n*= 784) and participants who consistently selected the same response on the EQ scale (*n*= 46). The intersection of these two (*n*= 21) were deemed to demonstrate a lazy response pattern. Using a chi-squared test, it was found that choosing the same EQ responses was associated with having zero contacts  (*χ*^2^ = 46.64, *p* < .001). We excluded the 46 participants who had consistently selected the same on the EQ scale and re-ran all analyses; the significances were all robust throughout.

### Comparing Alternate Modes of Contact: Messaging, Phone, and Video (H1)

A final output from the Poisson analysis reported in H1 showed that messaging was most common, followed by video, then phone, and finally FTF.

**Table SI1**

*Zero-parts Coefficients*

| Variable | Estimate | *EXP* | *SE* | *Z* | *p* |
| --- | --- | --- | --- | --- | --- |
| Message - FTF | 1.15 | 3.16 | 0.03 | 38.09 | <.001 |
| Phone – FTF | 0.62 | 1.86 | 0.03 | 19.90 | <.001 |
| Video - FTF | 0.77 | 2.17 | 0.03 | 24.74 | <.001 |
| Phone - message | -0.53 | 0.59 | 0.04 | -12.17 | <.001 |
| Video - message | -0.38 | 0.68 | 0.04 | -8.69 | <.001 |
| Video - phone | 0.15 | 1.16 | 0.04 | 3.42 | .001 |

### Gender and Age Have No Significant Effect on Modes of Contact

**Table SI2**

*Modes of Contact by Gender, Fixed Effects*

| Variable | Estimate | *EXP* | *SE* | *z* | *p* |
| --- | --- | --- | --- | --- | --- |
| Messaging | 0.82 | 2.27 | .06 | 14.23 | <.001 |
| Phone | 0.55 | 1.73 | .06 | 9.38 | <.001 |
| Video | 0.50 | 1.65 | .06 | 8.47 | <.001 |
| Gender | -0.02 | 0.98 | .06 | -0.34 | .732 |
| Total contacts | 0.76 | 2.14 | .01 | 64.10 | <.001 |
| Stringency | -0.08 | 0.92 | .01 | -7.06 | <.001 |
| Message * Gender | 0.44 | 1.55 | .07 | 6.43 | <.001 |
| Phone * Gender | 0.10 | 1.10 | .07 | 1.47 | .142 |
| Video * Gender | 0.36 | 1.43 | .07 | 5.12 | <.001 |

*Note:* Reference categories are FtF contact and gender (men).

**Table SI3**

*Modes of Contact by Age, Fixed Effects*

| Variable | Estimate | *EXP* | *SE* | *z* | *p* |
| --- | --- | --- | --- | --- | --- |
| Messaging | 1.11 | 3.03 | .07 | 15.52 | <.001 |
| Phone | 0.38 | 1.46 | .08 | 5.00 | <.001 |
| Video | 0.67 | 1.95 | .07 | 9.06 | <.001 |
| Age 25-34 | -0.06 | 0.94 | .09 | -0.63 | .526 |
| Age 35-44 | -0.05 | 0.95 | .10 | -0.56 | .573 |
| Age 45-54 | -0.17 | 0.84 | .10 | -1.60 | .109 |
| Age 55-64 | -0.09 | 0.91 | .11 | -0.82 | .412 |
| Age 65-90 | -0.15 | 0.86 | .12 | -1.16 | .247 |
| Total contacts | 0.78 | 2.18 | .01 | 65.03 | <.001 |
| Stringency | -0.09 | 0.91 | .01 | -7.34 | <.001 |
| Message * Age (25-34) | 0.13 | 1.14 | .09 | 1.39 | .163 |
| Phone * Age (25-34) | 0.23 | 1.26 | .10 | 2.49 | .013 |
| Video * Age (25-34) | 0.18 | 1.20 | .10 | 1.97 | .049 |
| Message * Age (35-44) | -.003 | 0.99 | .09 | -0.03 | .977 |
| Phone * Age (35-44) | 0.20 | 1.22 | .10 | 1.92 | .054 |
| Video * Age (35-44) | 0.14 | 1.15 | .10 | 1.42 | .155 |
| Message * Age (45-54) | 0.001 | 1.00 | .11 | 0.01 | .994 |
| Phone * Age (45-54) | 0.42 | 1.52 | .11 | 3.80 | <.001 |
| Video * Age (45-54) | 0.06 | 1.06 | .11 | 0.58 | 561 |
| Message * Age (55-64) | -0.10 | 0.90 | .11 | -0.86 | .387 |
| Phone * Age (55-64) | 0.33 | 1.39 | .12 | 2.85 | .004 |
| Video * Age (55-64) | 0.01 | 1.01 | .12 | 0.09 | .925 |
| Message * Age (65-89) | -0.08 | 0.92 | .13 | -0.64 | .524 |
| Phone * Age (65-89) | 0.44 | 1.55 | .13 | 3.37 | .001 |
| Video * Age (65-89) | -0.14 | 0.87 | .14 | -1.00 | .318 |

*Note:* Reference categories are FtF contact and age (16-25).

### Death Slope and Stringency Analyses

**Table SI4**

*Zero-parts Coefficients for the Death Slope Analyses*

| Variable | Estimate | *EXP* | *SE* | *Z* | *p* |
| --- | --- | --- | --- | --- | --- |
| Message | -1.67 | 0.19 | .10 | -15.90 | <.001 |
| Phone | -1.72 | 0.18 | .11 | -15.27 | <.001 |
| Video | -1.84 | 0.16 | .13 | -14.58 | <.001 |
| Death slope | 0.28 | 1.32 | .06 | 4.58 | <.001 |
| Message * death slope | -0.82 | 0.44 | .10 | -7.83 | <.001 |
| Phone * death slope | -0.48 | 0.62 | .11 | -4.25 | <.001 |
| Video * death slope | -0.90 | 0.41 | .13 | -6.82 | <.001 |

### Analysing Low Stringency Participants Only (H1)

**Table SI5**

*Low Stringency Participants Only: Modes of Contact by Staying Home, Fixed Effects*

| Variable | Estimate | *EXP* | *SE* | *z* | *p* |
| --- | --- | --- | --- | --- | --- |
| Messaging | 0.69 | 1.99 | .09 | 7.25 | <.001 |
| Phone | 0.32 | 1.38 | .10 | 3.20 | .001 |
| Video | 0.22 | 1.25 | .11 | 2.02 | .043 |
| Home (sometimes) | 0.06 | 1.06 | .12 | 0.46 | .647 |
| Home (yes) | -0.22 | 0.80 | .11 | -1.94 | .052 |
| Total contacts | 0.70 | 2.01 | .02 | 33.14 | <.001 |
| Stringency | 0.03 | 1.03 | .02 | 1.23 | .218 |
| Message * Home (sometimes) | 0.11 | 1.12 | .12 | 0.86 | .387 |
| Phone * Home (sometimes) | 0.10 | 1.10 | .13 | 0.71 | .476 |
| Video * Home (sometimes) | 0.12 | 1.13 | .14 | 0.81 | .418 |
| Message * Home (yes) | 0.46 | 1.58 | .12 | 3.95 | <.001 |
| Phone * Home (yes) | 0.35 | 1.42 | .12 | 2.93 | .003 |
| Video * Home (yes) | 0.50 | 1.65 | .13 | 3.86 | <.001 |

*Note:* Reference categories are FtF contact and home (no).

**Table SI6**

*Low Stringency Participants Only: Modes of Contact in Relation to FtF During Peak Restrictions and Easing of Lockdown Acceleration and Deceleration Phases of the Pandemic Death Slope), Fixed Effects.*

| Variable | Estimate | *EXP* | *SE* | *z* | *p* |
| --- | --- | --- | --- | --- | --- |
| Messaging | 0.93 | 2.53 | .05 | 20.48 | <.001 |
| Phone | 0.51 | 1.66 | .05 | 10.56 | <.001 |
| Video | 0.50 | 1.65 | .05 | 10.25 | <.001 |
| Death slope | 0.02 | 1.02 | .04 | 0.50 | .620 |
| Total contacts | 0.67 | 1.95 | .02 | 32.73 | <.001 |
| Gender | 0.37 | 1.45 | .04 | 8.67 | <.001 |
| Age | -0.05 | 0.95 | .02 | -2.22 | .026 |
| Home (sometimes) | 0.16 | 1.17 | .06 | 2.66 | .008 |
| Home (yes) | 0.16 | 1.17 | .05 | 3.05 | .002 |
| Education | 0.03 | 1.30 | .02 | 1.44 | .151 |
| Message * Death slope | -0.05 | 0.95 | .04 | -1.20 | .229 |
| Phone * Death slope | -0.02 | 0.98 | .04 | -0.56 | .578 |
| Video * Death slope | -0.06 | 0.94 | .03 | -1.46 | .145 |

*Note:* Reference categories are FtF contact and home (no).

### Replacing Death Slope for Stringency

**Table SI7**

*Modes of Contact in Relation to FtF During Peak Restrictions and Easing of Lockdown (Stringency), Fixed Effects.*

| Variable | Estimate | *EXP* | *SE* | *z* | *p* |
| --- | --- | --- | --- | --- | --- |
| Messaging | 1.14 | 3.13 | .03 | 32.71 | <.001 |
| Phone | 0.59 | 1.80 | .04 | 16.28 | <.001 |
| Video | 0.76 | 2.14 | .04 | 21.23 | <.001 |
| Stringency | -0.12 | 0.89 | .03 | -4.75 | <.001 |
| Total contacts | 0.74 | 2.09 | .02 | 58.64 | <.001 |
| Gender | 0.31 | 1.36 | .03 | 11.47 | <.001 |
| Age | -0.03 | 0.97 | .01 | -2.58 | .010 |
| Home (sometimes) | 0.01 | 1.01 | .04 | 0.18 | .855 |
| Home (yes) | 0.05 | 1.05 | .03 | 1.46 | .145 |
| Education | 0.08 | 1.08 | .01 | 5.97 | <.001 |
| Message * Stringency | 0.10 | 1.01 | .03 | 3.62 | <.001 |
| Phone * Stringency | 0.09 | 1.09 | .03 | 2.92 | .004 |
| Video * Stringency | 0.05 | 1.05 | .03 | 1.73 | .084 |

*Note:* Reference category is FtF contact.

### H2 Self- and Household Vulnerability

**Table SI8**

*Self-Vulnerability and FTF Do Not Interact*

| Variable | *B* | SE | *Beta* | *t* | *p* |
| --- | --- | --- | --- | --- | --- |
| Age | 0.80 | 0.07 | 0.19 | 12.19 | < .001 |
| Total contacts | 0.06 | 0.06 | 0.01 | 0.92 | .357 |
| Stringency | 0.71 | 0.06 | 0.17 | 11.54 | < .001 |
| Gender | -0.84 | 0.13 | -0.10 | -6.51 | < .001 |
| Home | -0.14 | 0.08 | -0.03 | -1.81 | .070 |
| Education | -0.08 | 0.09 | -0.01 | -0.84 | .401 |
| Self-vulnerability | -0.11 | 0.07 | -0.03 | -1.61 | .108 |
| FtF | 0.27 | 0.07 | 0.06 | 4.01 | < .001 |
| FTF  *  self-vulnerability | -0.10 | 0.06 | -0.03 | -1.62 | .106 |
| *Note:* *R^2^* = .07, *F*(9, 4534) = 37.32, *p* < .001. | | | | | |

**Table S19**

*Household Vulnerability and FtF Significantly Interact to Predict Wellbeing*

| Variable | *B* | *SE* | *Beta* | *t* | *p* |
| --- | --- | --- | --- | --- | --- |
| Age | 0.78 | 0.07 | 0.19 | 11.16 | < .001 |
| Total contacts | 0.09 | 0.07 | 0.02 | 1.24 | .215 |
| Stringency | 0.73 | 0.07 | 0.18 | 11.12 | < .001 |
| Gender | -0.91 | 0.14 | -0.10 | -6.57 | < .001 |
| Home | -0.15 | 0.08 | -0.03 | -1.86 | .063 |
| Education | -0.03 | 0.10 | -0.01 | -0.34 | .737 |
| Household vulnerability | -0.03 | 0.07 | -0.01 | -0.46 | .645 |
| FtF | 0.25 | 0.07 | 0.05 | 3.35 | < .001 |
| FtF  *  household vulnerability | -0.16 | 0.07 | -0.04 | -2.22 | .027 |

*Note:* DV = wellbeing. *R^2^* = .07, *F*(9, 4013) = 34.32, *p* < .001. We re-ran the model three times replacing FtF in the household vulnerability interaction for each DMC and the terms were not significant *(p’s* > .265).

**Figure SI1**

*People with More FtF Contact (+1SD) Tend to Have Greater Wellbeing Than People with Little FtF (-1SD), Especially When They Do Not Perceive Anyone in their Household as Vulnerable (-1SD).*

### Analyses within the Non-binary Sample

**Table S20**

| *FtF versus CMC within the Non-binary Sample* | | | | |
| --- | --- | --- | --- | --- |
| *Non-zero part coefficients* | | | | |
| Variable | Estimate | *SE* | *Z* | *p* |
| Message - FTF | 1.86 | 0.40 | 4.71 | <.001 |
| Phone – FTF | 1.06 | 0.41 | 2.59 | .009 |
| Video - FTF | 1.24 | 0.41 | 3.02 | .003 |
| *Zero part coefficients* | | | | |
| Variable | Estimate | *SE* | *Z* | *p* |
| Message - FTF | -3.85 | 4.32 | -0.89 | .37 |
| Phone – FTF | -1.09 | 1.34 | -0.81 | .42 |
| Video - FTF | -1.19 | 1.47 | -0.81 | .42 |

We replicate the findings with men and women by showing that non-binary people were more likely to use CMC over FtF when non-zero part coefficients are considered, but not when zero-part efficients are considered.

**Table S21**

*Wellbeing and each FtF and CMC mode (separate models) within the non-binary sample*

| Variable | Estimate | *SE* | *t* | *p* |
| --- | --- | --- | --- | --- |
| FtF | 0.68 | 0.90 | 0.76 | .45 |
| Message | .26 | .15 | 1.72 | .09 |
| Phone | .28 | .43 | 0.66 | .51 |
| Video | .36 | .26 | 1.39 | .17 |

**Figure SI2**

*Wellbeing and each FtF and CMC mode within the non-binary sample*

*
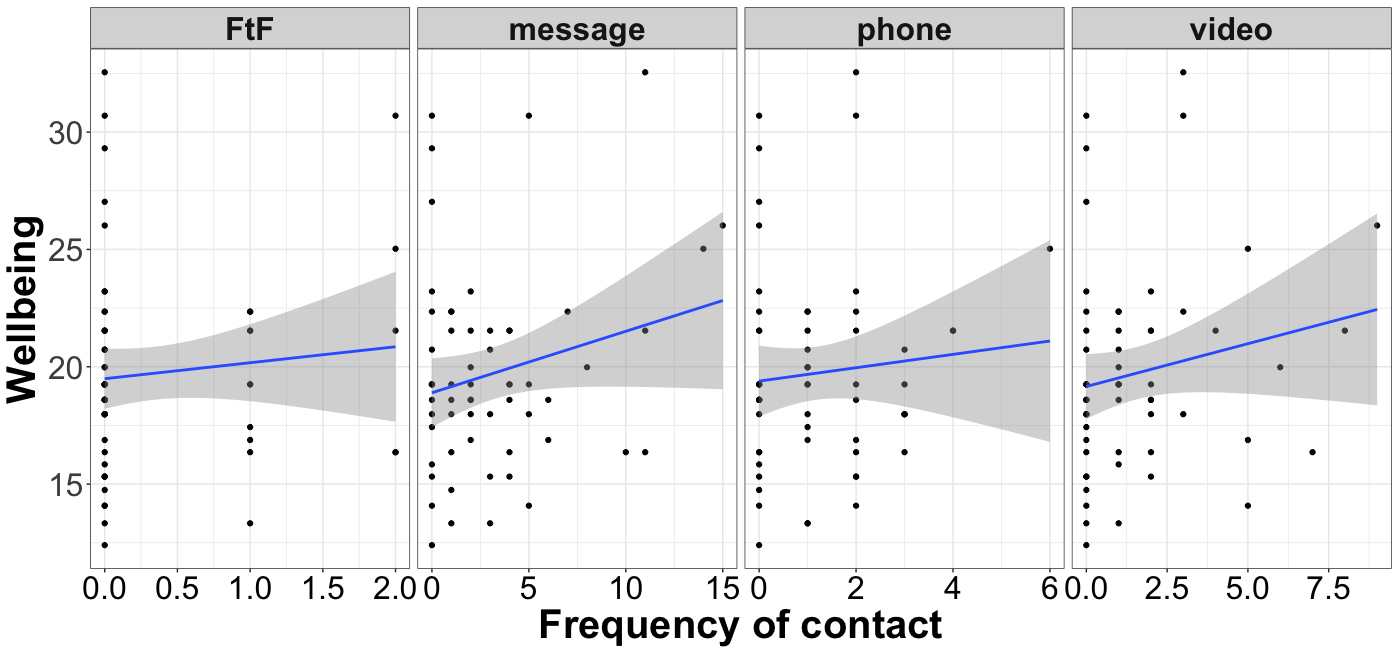
*

**Table S22**

*Wellbeing, self-vulnerability and FtF versus CMC within the non-binary sample*

| Variable | *B* | SE | *t* | *p* |
| --- | --- | --- | --- | --- |
| Age | 2.14 | 0.78 | 2.75 | .008 |
| Total contacts | 0.60 | 0.73 | 0.82 | .42 |
| Stringency | 0.87 | 0.60 | 1.45 | .15 |
| Home (no vs yes) | 1.83 | 1.64 | 1.12 | .27 |
| Education | -0.45 | 0.80 | -0.57 | .57 |
| Self-vulnerability | 0.002 | 0.03 | 0.05 | .96 |
| FtF | 4.50 | 1.98 | 2.27 | .03 |
| FTF  *  self-vulnerability | -0.08 | 0.04 | -2.03 | .05 |

**Table S23**

*Wellbeing, household vulnerability and FtF versus CMC within the non-binary sample*

| Variable | *B* | SE | *t* | *p* |
| --- | --- | --- | --- | --- |
| Age | 2.14 | 0.86 | 2.47 | .02 |
| Total contacts | 0.31 | 0.87 | 0.36 | .72 |
| Stringency | 0.18 | 0.74 | 0.25 | .81 |
| Home (no vs yes) | 1.32 | 1.83 | 0.72 | .48 |
| Education | -0.62 | 0.92 | -0.67 | .51 |
| Household vulnerability | 0.002 | 0.03 | 0.05 | .96 |
| FtF | 4.50 | 1.98 | 2.27 | .03 |
| FTF  *  household vulnerability | -0.08 | 0.05 | -1.63 | .11 |

## SI References

Baron-Cohen S, and Wheelwright S (2004) The empathy quotient: an investigation of adults with Asperger syndrome or high functioning autism, and normal sex differences. *Journal of autism and developmental disorder,*34(2), 163-175. DOI:10.1023/b:jadd.0000022607.19833.00

Stewart-Brown S, Tennant A, Tennant R, Platt S, Parkinson J and Weich S (2009) Internal construct validity of the Warwick-Edinburgh mental well-being scale (WEMWBS): a Rasch analysis using data from the Scottish health education population survey. *Health and quality of life outcomes*7(1): 1-8.
DOI:10.1186/1477-7525-7-15

Taggart F, Stewart-Brown S, and Parkinson J (2015) Warwick-Edinburgh Mental Well-Being Scale (WEMWBS), User Guide Version 2 May 2015. Available at https://studylib.net/doc/12455513/warwick-edinburgh-mental-well-being-scale--wemwbs--user-g... (accessed 06.05.2021)

Muncer SJ and Ling J (2006) Psychometric analysis of the empathy quotient (EQ) scale. *Personality and Individual differences* 40(6): 1111-1119. DOI:10.1016/j.paid.2005.09.020
